# Overall survival and quality of life in elderly patients treated for rectal cancer: results of a five year follow-up

**DOI:** 10.1186/1471-2482-13-S1-A40

**Published:** 2013-09-16

**Authors:** Giovanni Romano, Francesco Moccia, Alfredo Allaria, Gianluca Rossetti, Domenico Napoletano, Beniamino Pascotto, Marco Cimmino, Francesco Orlando, Maria Chiara Bondanese, Landino Fei

**Affiliations:** 1 Division of Gastrointestinal Surgery, Department of Anaesthesiological, Surgical and Emergency Sciences, Second University of Naples-School of Medicine, Via Pansini 5, 80131 Naples, Italy

## Background

The study was conducted on a population of 74 patients undergoing surgery for rectal adenocarcinoma with 5-year follow-up. The aim is to evaluate the survival and Quality of Life (QoL) in two groups of patients: Y group with age <60 years and E group with age ≥ 60 years. QoL was determined by administration of C30 Quality of Life Questionnaire (QLQ C30) and the module ColoRectal-29 (CR-29) [1].

## Methods

By analyzing some surgical cases in our Gastrointestinal Surgery Division, we have selected 74 subjects operated for rectal cancer, during year 2002 and 2007. They completed a 5 year follow-up. The sample consisted of 45 men and 29 women with a mean age of 62 ± 10 years (range 28-84). We have formed two groups according to their age: the Y group of 34 patients aged <60 years and the E group of 40 patients aged ≥ 60 years.

Fifty-eight patients had an anterior resection of the rectum with packaging of low or ultra-low anastomosis, while 11 patients were operated according to the Miles’s abdomino-perineal amputation technique and 5 had a Hartmann’s rectal surgery. All the patients contributed to the study by signing a specific informed consent. We calculated the percentage of an overall survival at 5 years after surgery while the impact of the neoplastic disease on the life quality of the patients affected by rectal cancer has been evaluated through specific questionnaires, widely used in scientific literature[2-4]: the QLQ C-30 and CR-29, developed by the European Organization for Research and Treatment of Cancer (EORTC) which consists in 59 questions. These modules include several questions grouped macro-topics called "dimensions" of evaluation. Each dimension of functional assessment has a score enclosed from 0 to 100. The highest score detected in every single "dimension" corresponds to the best health condition of the patient. The numerical value 50 represents the cut-off between a good value and a mediocre one.

The presentation of the questionnaire was carried out by a blinded external observer so that any interpretative bias could be avoided.

## Results

The overall survival rate was 61%. In agreement with the study published by Ponz De Leon et al.[2] and analyzing the survival’s results, corrected for the independent variable age, it becomes clear that the prognosis is significantly better in patients under the age of 60 (Y group = 85%; E group = 46%).

The average scores of the questionnaire QLQ C-30 and CR-29, show values up to 50 in all functional scales both in the Y group and the E group. In particular, scores are high in emotional functions (Y group: 84 ,19; E group: 73,6) and in cognitive ones (Y group: 93,1; E group: 87,2). The Global Health Perception value (GHP) obtained is quite comparable between the two groups (Y group: 65,8; E group: 66,1). The Role Physical score (RP), which assesses the physical status comparing to the activity carried out daily by the patient (work, hobbies ... etc), is the only one to be less than 50 but only in the Y group (RP = 37,4); while the value of RP in the E group turns out to be 50,4. This result could be explained by considering that the sample of younger patients, examined before the cancer diagnosis, initially performed a regular work which was partially limited by the disease at a later stage. The major discomforts found out in our sample are determined, in both groups, by the presence and management of the stoma (Y group: 60,5; E group: 52,6).

## Conclusions

The results of our case study were compared with some further studies [2,3] but considering the same period of follow-up. They showed a steady increase in the survival rate since 1978, probably related to the different historical period and to the availability of different cutting-edge technologies and innovative medicines. The best prognosis, detected in subjects under 60 might be due to a greater attention that younger patients with a family history of colorectal neoplasia give to symptoms even when they’re blurred. They usually have screening tests earlier than the general population and are physically more suitable to undergo to a more radical surgery.

The results of the questionnaire QLQ C-30 indicate a good general quality of life in both groups. When it comes to the evaluation of the quality/quantity of discomforts, our studies underline that the main problems effecting the life quality of the patients are the presence/ management of the permanent stoma (peri-stomal skin irritation, alteration of body image and reduction of social activities) as well as all the collateral effects of the neo-/adjuvante treatment (alopecia, anorexia and weight loss, gastrointestinal symptoms ) and other nonspecific symptoms which are not directly related to the surgical treatment.

According to the definition of the O.M.S. health is a state of "complete physical, mental and social well-being and not merely the absence of disease or infirmity". Nowadays, the questionnaires for the assessment of the quality of life represent a valuable tool to measure how serious diseases negatively affect the patients’ daily life. The results obtained after 5 years from a rectal cancer surgery are very encouraging in terms of both survival and general quality of life. Moreover all the discomforts occurred do not seem to influence significantly the evaluation of the quality of life of the patients who were interviewed.

The real advantage of the questionnaires on quality of life and survival studies are more evident in researches with a long follow-up [4]. Our future goal will be to improve the current survey through the expansion of cases regarding the 5-year follow-up and the re-evaluation of the group of patients examined in this study with 10-year follow-up.
